# Classification of Chemotherapy-Induced Febrile Neutropenic Episodes Into One of the Three Febrile Neutropenic Syndromes

**DOI:** 10.7759/cureus.42843

**Published:** 2023-08-02

**Authors:** Ronak Raheja, Neelesh Reddy, Twinkle Patel, Srikar Kilambi, Ashik A Mathew, Abdul Majeed

**Affiliations:** 1 Department of Internal Medicine, Kempegowda Institute of Medical Sciences, Bengaluru, IND; 2 Department of Medical Oncology, Columbia Asia Referral Hospital Yeshwanthpur, Bangalore, IND; 3 Department of Internal Medicine, Shri Sathya Sai Medical College and Research Institute, Surat, IND; 4 College of Medicine, Sri Ramachandra Institute of Higher Education and Research, Chennai, IND; 5 Department of Pharmacology and Therapeutics, Manipal Hospitals, Bangalore, IND; 6 Department of Internal Medicine, Columbia Asia Referral Hospital Yeshwanthpur, Bangalore, IND

**Keywords:** neutropenic syndromes, chemotherapy-induced neutropenia, culture sensitivity, cause of fever, correlation, blood culture, clinical presentation, febrile neutropenia, neutropenia

## Abstract

Introduction

Febrile neutropenia is a commonly encountered medical emergency in patients undergoing cancer treatment and can delay and modify the course of treatment and even lead to dire outcomes, including death. The cause of fever in a post-chemotherapy-induced neutropenic patient can be confusing to treating physicians. A review of the literature demonstrated that blood culture results could determine the cause of febrile neutropenia in only approximately 10% to 25% of patients. The objective of our study was to measure the incidence of positive blood cultures, urine cultures, and other body fluid cultures resulting in chemotherapy-induced neutropenia and further classify fever episodes into three neutropenic fever syndromes, such as microbiologically documented, clinically suspected, or unknown causes of fever, respectively.

Methods

We conducted a prospective observational study on 399 chemotherapy-induced neutropenic fever episodes with the aim of classifying them into one of the three neutropenic syndromes. We tried to document the cause of the fever in these patients. We also noted the type of cancer treatment regimen they were on and correlated their clinical profile with their body fluid cultures, including blood cultures, urine cultures, and other body fluid cultures. We then categorized each fever episode into one of three neutropenic syndromes.

Results

We studied 399 febrile neutropenic episodes. We were able to microbiologically document the cause of fever in 39% of the cases, and we obtained growth in 51 out of 399 blood cultures (13%), which was comparable to the available literature, and urine culture showed growth in 62 out of 399 cultures (16%), while other body cultures such as pus culture, bile culture, and bronchioalveolar lavage cultures collectively showed growth in 42 out of 399 episodes (10%). The most common bacteria isolated in both blood and urine cultures were *Escherichia coli*. Cumulatively, including blood, urine, and body fluid cultures, we were able to classify 39% (155 out of 399 cases) of febrile neutropenic episodes as microbiologically documented. The cause of fever was clinically suspected by means of careful history taking and an extensive physical examination in 31% (125 out of 399) without growth evidence in blood cultures, urine cultures, or any other body fluid culture. The cause of fever remained unknown in 119 cases (30%) of patients and was classified under the unknown cause of fever.

Conclusions

We conclude by stating that the study of fever in a neutropenic patient should include a thorough history and clinical evaluation of blood, urine, and other body fluid cultures instead of solely relying on blood culture results. We recommend further classifying patients into one of the three neutropenic fever syndromes, such as those that are microbiologically documented, clinically suspected, or unknown. Our blood cultures were able to give us a 13% positivity rate, whereas microbiologically, we were able to isolate an organism likely causing fever in 39% of patients. The cause of fever was suspected clinically in 31% of patients, but we were unsuccessful in microbiologically documenting any culture growth in blood, urine, or any other body fluid culture. The cause of fever remained a mystery and unknown to us without any microbiological or clinical cues in 119 cases (30%) of febrile neutropenic episodes.

## Introduction

Chemotherapy-induced neutropenia is characterized by a reduction in neutrophil counts, usually occurring within 7 to 12 days following cancer chemotherapy. Febrile neutropenia is a concerning complication of chemotherapy and a major cause of morbidity, with compromised efficacy resulting from delays and dose reductions in chemotherapy. The cause of fever in chemotherapy-induced neutropenic patients has always been a debated topic of discussion. Understanding, identifying, and proving the cause of fever has always been confusing for treating physicians. The presence of central lines and chemotherapy ports further complicates the approach. Some authors believe cancer patients receiving chemotherapy, which adversely affects cellular mitosis, myelopoiesis, and the developmental integrity of the gastrointestinal mucosa and other epidermal cell surfaces, are at risk for invasive infection due to colonizing or pathogenic bacteria and/or fungi that translocate across mucosal surfaces.

The possibility of bloodstream infection is a major concern at the onset of fever since blood culture isolates account for only 10-25% of all febrile episodes in neutropenic patients [1]. These results indicated that the cause of fever in approximately 75% of neutropenic patients was uncertain or unclearly documented and documented as a fever of unknown origin. Some experts believe the patient's endogenous flora could be the source of infection due to breaks in mucosal surface integrity in up to 80% of cases [2]. While others believe that blood cultures alone are sufficient, unfortunately, the positive yield of blood cultures decreases further in neutropenic patients, thereby indicating a more structured and systematic study of other body fluid cultures and any clinical focus like central lines and chemo ports and then the classification of neutropenic fevers into one of three clinical syndromes based on culture results.

Researchers found that the occurrences of severe sepsis and septic shock in the setting of febrile neutropenia have been estimated to be, respectively, 20-30% and 5-10% [3]. Available literature showed a range of 10-25% of febrile neutropenic episodes in which bacteria were isolated as the cause of fever on the basis of the evaluation of blood cultures alone [4].

The following observations have been made about bacterial infections in neutropenic patients:

Epidermidis is the most common gram-positive pathogen, accounting for approximately one-half of all infections due to gram-positive infections. It is much less virulent than other bacterial pathogens, is frequently a contaminant, and can misguide the treatment approach [5].

Polymicrobial infections are rare, but their frequency appears to be rising.

Evaluation of the date of insertion of catheters, tubes, and lines along with a general examination around the central line entry point for tenderness or signs of possible infection like pus pockets can help a clinician evaluate and approach an episode of fever in a neutropenic patient, especially in between chemotherapy cycles. It is also worthy of mention that fevers that occur when fluids are given through a particular line or cannula could possibly be due to infection of the venous access. A decrease in the intensity or frequency of fever spikes after stopping the use of a central line could be suggestive of a possible infection of the line. It is also useful to send cultures from the venous port and another site. A possible antibiotic lock could be attempted to clear up the infection from the line, but in advanced infections, we may need to remove the line in view of a possible clinical focus of infection.

The Immunocompromised Host Society has classified initial neutropenic fever syndromes into the following three categories:

1) Microbiologically documented infection: neutropenic fever with a clinical focus on infection and an associated pathogen on cultures.

2) Clinically documented infection: neutropenic fever with a clinical focus (e.g., cellulitis, pneumonia), but without the isolation of an associated pathogen.

3) Unexplained fever: neutropenic fever with neither a clinical focus on infection nor an identified pathogen.

The classification and correlation of clinical presentations and microbiological manifestations help a clinician evaluate and approach an episode of fever in a neutropenic patient. We wanted to observe, classify, and compare the global literature on a series of 399 febrile neutropenic patients and classify the resultant trends into one of the three neutropenic syndromes.

## Materials and methods

A prospective observational study was conducted over a period of 21 months, from September 2019 to June 2021. The study was conducted at Columbia Asia Referral Hospital Yeshwanthpur oncology clinic and involved 399 chemotherapy-induced neutropenic patients, including 219 hematological malignancies and 180 solid tumors. Febrile neutropenia was diagnosed in the occurrence of a single oral temperature of ≥38.3°C (101°F) or 38.0°C (100.4°F) for more than one hour along with an absolute neutrophil count (ANC) ≤500/µl or ≤1000/µl with a predicted rapid decline during the next 48 hours. The ANC was calculated by looking at the differential white cell count or by multiplying the total white cell count by * percentage of neutrophils in the differential count.

All patients underwent a comprehensive clinical examination to assess indications of infection and potential source of infection. Additionally, blood, urine, and other body fluids were collected for aerobic and anaerobic cultures. Patients were given empirical antibiotic monotherapy within one hour of presentation with a broad-spectrum agent like cefepime, piperacillin-tazobactam, ceftazidime, or a carbapenem (meropenem, imipenem). The type of cancer treatment regimen and correlation of their clinical profile with their blood culture results, urine culture results, and other body fluid culture results were noted and then further classified into one of the three neutropenic fever syndromes as microbiologically documented, clinically suspected, or unknown causes of fever. The trial was conducted in accordance with the Declaration of Helsinki and all applicable national and local ethical requirements.

Inclusion criteria

The inclusion criteria include all patients attending Columbia Asia Referral Hospital Yeshwanthpur oncology clinic with febrile neutropenia.

Exclusion criteria

The exclusion criteria include all non-oncology febrile neutropenia and febrile neutropenic patients with COVID-positive status.

## Results

Classification of febrile neutropenia episodes into neutropenic syndromes

**Figure 1:**
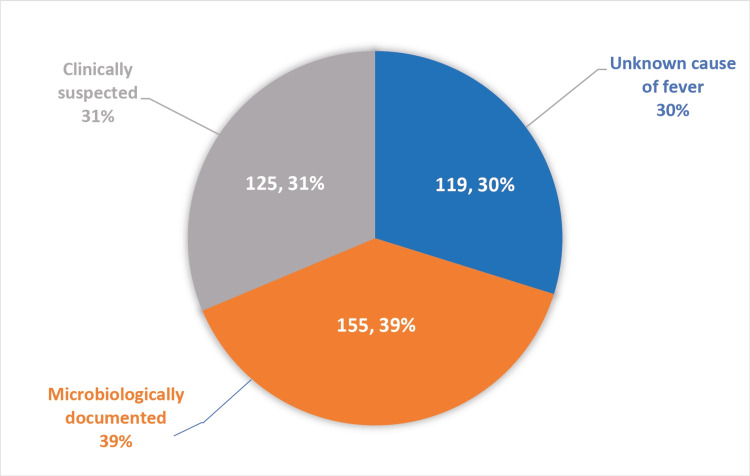
Classification of neutropenic fevers into neutropenic syndromes Microbiologically documented cases include positive growth on blood culture, urine culture, pus culture, bile culture, or any other body fluid culture. Clinically suspected cases are those with clinical evidence in either history or physical examination but who fail to provide any growth in body cultures. Unknown causes of fever are those cases of neutropenic fever with neither a clinical focus on infection nor an identified pathogen.

A total of 399 cases of febrile neutropenia were subclassified into neutropenic syndromes. Microbiologically documented cases are those neutropenic fevers in which a bacterium has been successfully cultured and demonstrated. Clinically suspected cases are those in which we found clinical signs of infection but were unable to culture any organism. Unknown cases are those in which the patients were febrile without any clear-cut clinical reasoning or microbiological proof. The results of our study are shown below in tabular format in Table 1 and in graphical format in Figure 1 for ease of reading and interpretation.

**Table 1:** Classification of neutropenic fevers into neutropenic syndromes

Neutropenic fever syndrome	Cases	Percentage
Microbiologically documented	155	39
Clinically suspected	125	31
Unknown	119	30
Total	399	100

Blood cultures

**Figure 2:**
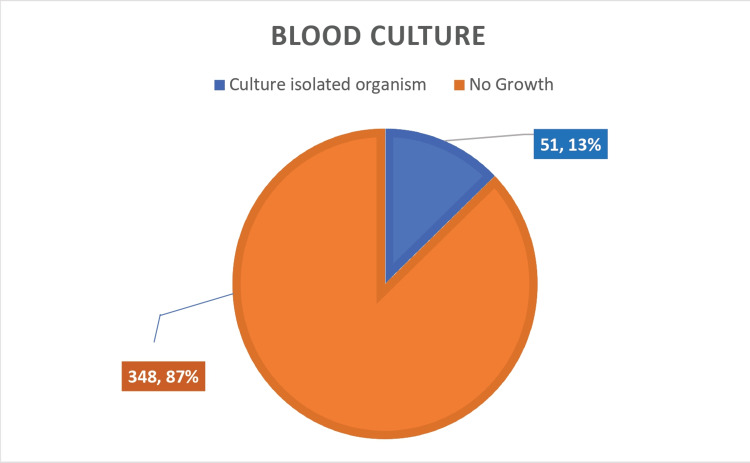
Blood culture result in febrile neutropenic patients

A total of 399 blood cultures from neutropenic patients who were febrile were collected. The number of positive blood cultures was 51 (13%) with a positivity rate as shown in graphical format in Figure 2 below.

Blood culture organism trends

**Figure 3:**
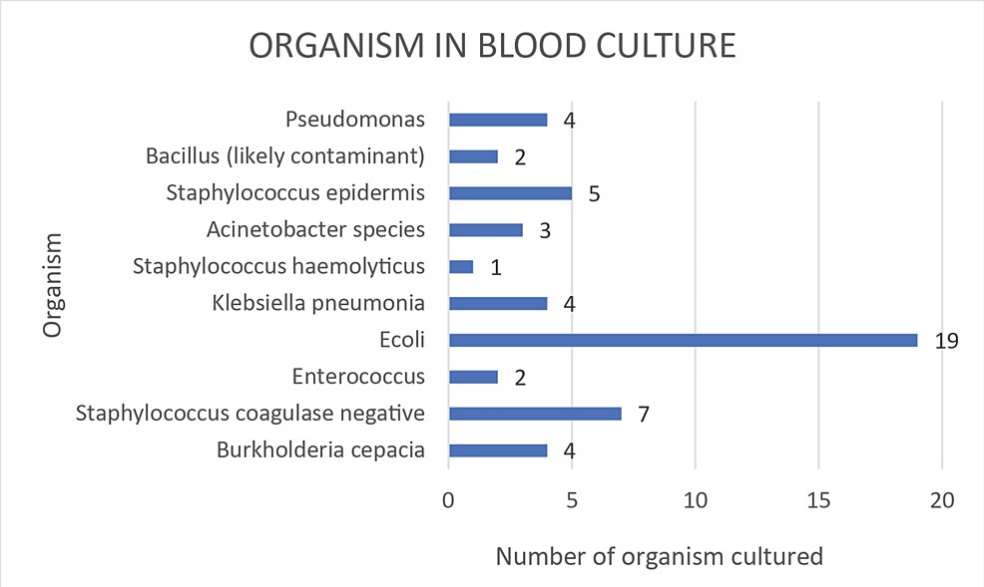
Organism trends in blood culture E. coli: Escherichia coli

Bacteremia was identified in 51 cases out of 399 patients' blood cultures, with a blood culture-only positivity rate of 13%. *E. coli* was the most commonly cultured organism, with 19 cases. In context with the demonstrated literature, the next most cultured group was the coagulase-negative staphylococcus group, with seven cases. *Staphylococcus epidermidis* was the next to follow with five cases. The trends in blood culture organisms are shown in graphical format in Figure 3 below.

Urine cultures

**Figure 4:**
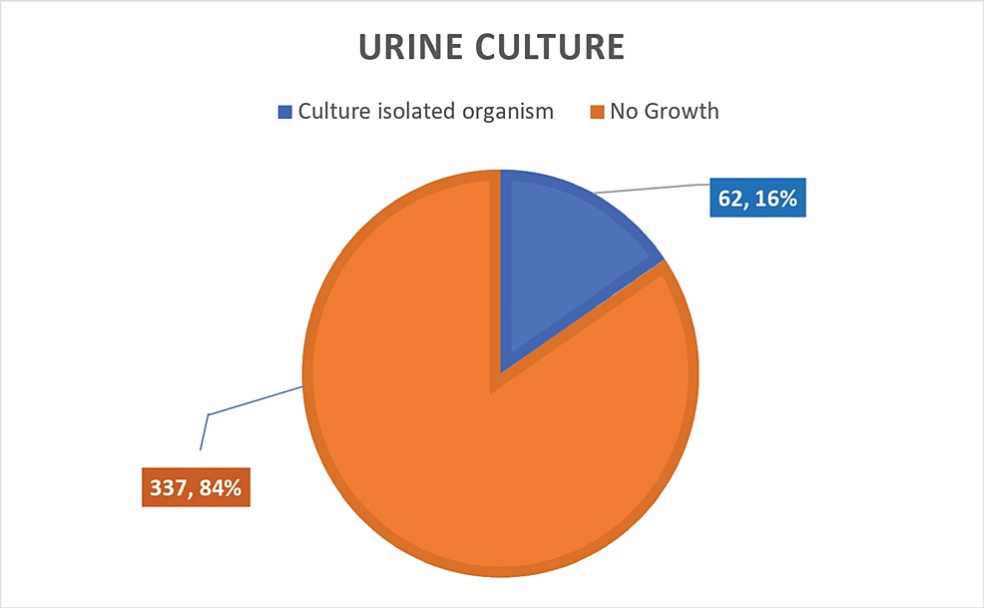
Urine culture results in febrile neutropenic patients

A total of 399 febrile neutropenic episodes were studied. We obtained positive urine cultures in 62 cases, with a 16% positivity rate. We obtained a negativity rate of 84% in urine cultures as well, as shown in graphical format in Figure 4 below.

Urine culture organism trends

**Figure 5:**
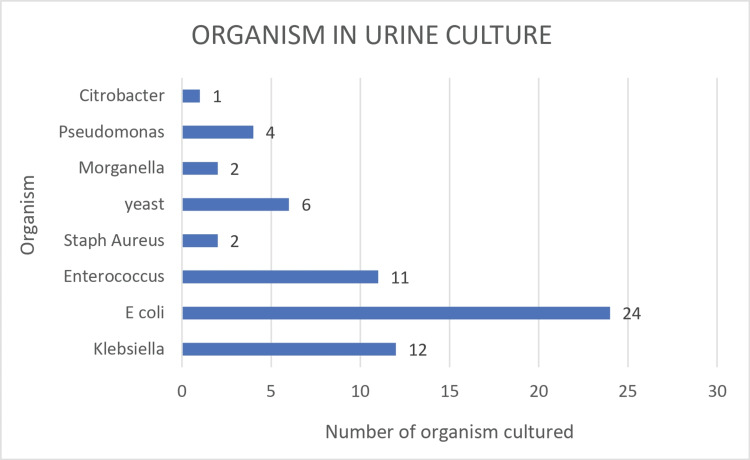
Organism trends in urine culture *E. coli*: *Escherichia coli*

Bacteremia was identified in 62 cases out of 399 patients' urine cultures, with a urine culture-only positivity rate of 16%. *E. coli* was the most commonly cultured organism, with 24 cases. The next most cultured group was the *Klebsiella* group, with 12 cases. *Enterococcus* was next to follow with 11 cases. The trends in urine culture organisms are shown in graphical format in Figure 5 below.

## Discussion

Patients usually follow up in the outpatient clinic a few days after chemotherapy administration. Clinical signs and symptoms that could predict the development of severe neutropenic complications are listed below:

a) Septic appearance b) Severe mucositis c) Intravascular catheter in-situ and suspected site of infection d) possibility of lower respiratory tract infection in chronic obstructive pulmonary disease e) Uncontrolled or progressive cancer

Bacteremia was identified and confirmed by blood cultures as a source of infection in 51 (13%) patients, which approximately correlated to the available literature.

We were able to microbiologically document the source of fever in an additional 26% of patients by collectively looking at all body fluid cultures. The International Immunocompromised Host Society neutropenic syndrome classification is listed in Table 2 below.

**Table 2:** International Immunocompromised Host Society neutropenic syndrome classification

Neutropenic syndrome classification	Explanation
Microbiologically documented infection	Neutropenic fever with a clinical focus on infection and an associated pathogen on cultures (blood, urine, or other body fluid cultures)
Clinically documented infection	Neutropenic fever with a clinical focus determined through history and examination (e.g., cellulitis, pneumonia) but without the isolation of an associated pathogen on any body fluid culture
Unexplained fever	Neutropenic fever with neither a clinical focus of infection nor an identified pathogen on any blood culture

Our blood culture positivity rate of 13% was comparable to the study done by Babu et al. at Kidwai Hospital, in which 15% of cases isolated and cultured an identifiable organism [6].

A review of the literature shows surprisingly low levels of positive blood cultures in febrile neutropenic patients. The possible causes for the same could be listed below:

1) Missing the organism 2) Systemic inflammatory response syndrome v/s sepsis [7] 3) Viral, fungal, or protozoal pathogens that are missed on standard aerobic cultures [8] 4) Prior antibiotic administration in the last 48 hours [9] 5) Miscellaneous, unidentified metabolic causes like thyrotoxicosis and malignant hyperthermia [10] 6) Intermittent bacteremia [11] 7) Culture-negative sepsis

Treatment of febrile neutropenia

Each cancer center should have an updated standard regimen of choice, which contributes to the utility of this study. The following points are to be considered when dealing with febrile neutropenic patients:

In culture-positive patients, we change the antibiotic to the most susceptible option based on the culture plate resistance pattern, as we are dealing with a bacterial pathogen. Broad-spectrum antibiotics should be given as early as possible, with recommendations to give antibiotics in the first 60 minutes and at full doses, adjusted for renal and/or hepatic function [12]. It is clear that in the intensive care setting, early administration of appropriate antibiotic therapy has a beneficial impact on sepsis [13]. Antibiotics chosen should be broad-spectrum and cover Gram-positive bugs, Gram-negative bugs, and polymicrobial infections [14]. Monotherapy is initiated with a broad-spectrum agent like cefepime, piperacillin-tazobactam, ceftazidime, or a carbapenem (meropenem, imipenem). Fluoroquinolones can be added in complicated patients and are used more for prophylaxis in high-risk cases than the initial antibiotic regimen.

Vancomycin is chosen after clinical evaluation if a skin-based infection like methicillin-resistant *Staphylococcus aureus*, community-acquired pneumonia, or severe mucositis is suspected. Vancomycin is also chosen as the first regimen for hemodynamic instability [4]. Linezolid is a good alternative to vancomycin and has shown equal efficacy. Anaerobic coverage with clindamycin or metronidazole can be considered in necrotizing mucositis, sinusitis, periodontal cellulitis, perirectal cellulitis, intra-abdominal infection (including neutropenic enterocolitis [typhlitis]), and pelvic infection. Combination therapy is preferred as an empiric choice but has not been shown to be any better than any broad-spectrum monotherapy [15].

In culture-negative sepsis, we recommend the concept of de-escalation of antibiotics in clinically stable patients [16].

We follow the following stages of antibiotic administration in febrile patients admitted to the hospital:

Stage 1: Early administration of broad-spectrum antibiotics to get blanket coverage (before blood culture results have arrived) Stage 2: Focused, targeted therapy based on the culture sensitivity profile (after the arrival of blood culture results) Stage 3: Procalcitonin can be used as an adjunct to deescalate antibiotics, and literature has demonstrated that tracking procalcitonin improves mortality [17] Stage 4: We adhere to the three Ds of antibiotic stewardship: right duration, right dose, and de-escalation

Prophylaxis

Generally, prophylactic antibiotics are recommended only for patients expected to have fewer than 100 neutrophils/μL for more than seven days [18]. With increasing bacterial resistance, the benefit of continuous prophylactic fluoroquinolone therapy during prolonged neutropenia remains to be confirmed [1]. For example, levofloxacin 500 mg could be used continuously. Management of febrile neutropenia indicates antibiotic prophylaxis with an attempt to cover the most culture-resistant strain at a given center, but it is stepped down after 72 hours if no multidrug-resistant pathogen is isolated. Antibiotic stewardship and infection control programs are mandatory in every cancer center [4].

The Current National Comprehensive Cancer Network guidelines recommend consideration of bacterial prophylaxis with fluoroquinolone in intermediate- and high-risk patients [19]. The Infectious Diseases Society of America and the American Society of Clinical Oncology both recommend antibacterial prophylaxis with a fluoroquinolone in patients who are expected to remain profoundly neutropenic (with an ANC <100 cells/mm3) for more than seven days [20].

Antifungal therapy

Consider antifungal prophylaxis with fluconazole-like medicine for either an obvious fungal infection determined by imaging or a clinically unexplained persistent or recurrent fever if it lasts for more than four to seven days. Checking for fungal markers in the serum, such as the *Aspergillus* galactomannan antigen and the beta-D-glucan assay, should also be considered in high-risk patients. If the fever is persistent, recommendations direct us toward antifungal medications like voriconazole, echinocandins, or amphotericin-B [21].

Colony stimulating factors

Although most neutropenic patients are treated with colony-stimulating factors, the recommendations do not recommend the use of colony-stimulating factors like C-GSF. For catheter removal, the central venous catheter should also be removed in patients with complicated infections [22].

Anaerobes in febrile neutropenia

Anaerobic bacteria are abundant in the alimentary tract, but they are rare pathogens isolated from patients with neutropenic fever. This could be because blood cultures require separate aerobic and anaerobic tubes. Anaerobes could be the cause of necrotizing mucositis, periodontal cellulitis, mucositis, neutropenic enterocolitis, and intraabdominal and pelvic infections, but they have not been cultured frequently.

Treatment failure

No fever defervescence within 30 days of the start of treatment or persistence, progression, or recurrence of signs and symptoms of infection despite initial broad-spectrum therapy were considered treatment failures.

We do not have the exact documented rate of treatment failures and deaths, but a general observation we made in keeping with the available literature was that poor baseline status of the patients led to more severe infections where patients were escalated to the intensive care unit, where they were lost to follow-up [23].

The treatment failure rate is higher in documented infections than in unknown febrile episodes [24]. Treatment failure was seen more in patients with hematological cancers than in solid tumors. The various terms used in relation to our study are described in Table 3 below.

**Table 3:** Terminology and definitions used in the study ANC: absolute neutrophil count

Terminology	Description
Febrile neutropenia	Fever in a neutropenic patient
Persistent neutropenic fever	If the patient remains febrile for more than five days despite antibiotic therapy in hematological malignancies or more than two days despite adequate antibiotic therapy in solid tumors
Neutropenia	ANC <1500 or 1000 cells/mm3
Mild neutropenia	(1000 <= ANC < 1500): minimal risk of infection [3]
Moderate neutropenia	(500 <= ANC < 1000): moderate risk of infection
Severe neutropenia	Neutrophil count of <500 cells/mm3 or a count of <1000 cells/mm3 with a predicted decrease to <500 cells/mm3 within the next 48-72 hours [4]
Agranulocytosis	ANC is less than 200 cells/microliter
Chronic neutropenia	Neutropenia which lasts for longer than three months
Isolated neutropenia	Only a decrease in neutrophil counts with normal red blood cell counts and normal platelet counts
Granulocytopenia	Decrease in granulocyte counts, granulocytes compromise neutrophils, eosinophils, and basophils
Leukopenia	When total white blood cell counts are less than 4400 cells/microliter

## Conclusions

Our study included 399 febrile neutropenic episodes. We obtained growth in 51 out of 399 blood cultures (13%), and urine cultures showed growth in 62 out of 399 cultures (16%), whereas other body cultures like pus culture, bile culture, and bronchoalveolar lavage cultures collectively showed growth in 42 out of 399 episodes (10%). In all, we were able to microbiologically document the cause of fever in 155 out of 399 cases (39%), and the cause of fever was clinically suspected in 125 cases (31%). The cause of fever was unknown in 119 (30%) of the patients. About 219 episodes of febrile neutropenia were seen in hematological malignancies, whereas 180 episodes belong to advanced solid tumors. The most common bacteria isolated in both blood and urine cultures were *Escherichia coli*.

We conclude by recommending a thorough clinical examination and review of other bodily fluid cultures in febrile neutropenic patients, as we found a positivity of 13% in blood cultures alone but a positivity of 39% in microbiologically documented infections (including urine and other body fluid cultures). We recommend the classification of neutropenic patients into neutropenic syndromes, such as those that are microbiologically documented, clinically suspected, or of unknown cause. The process of classification helped us approach and manage neutropenic patients better.
